# COVID-19 vaccine hesitancy in Ethiopia in 2021: a multicenter cross-sectional study

**DOI:** 10.1016/j.ijregi.2022.11.006

**Published:** 2022-12-08

**Authors:** Besfat Berihun Erega, Wassie Yazie Ferede, Fillorenes Ayalew Sisay, Gebrehiwot Ayalew Tiruneh, Abeba Belay Ayalew, Erean shigign Malka, Habtamu Abie Tassew, Asrat Alemu

**Affiliations:** a Department of Midwifery, College of Medicine and Health Sciences, Debre Tabor University, Ethiopia; b Department of Midwifery, College of Medicine and Health Sciences, Selale University, Ethiopia; c Department of Midwifery, College of Medicine and Health Sciences, Dilla University, Ethiopia

**Keywords:** Vaccine hesitancy, COVID-19, multicenter study, Ethiopia

## Abstract

**Introduction:**

Worldwide, hesitancy to be immunized against SARS-CoV-2 is the most common barrier to reducing COVID-19 incidence. Our study investigated determinants for hesitancy and will be helpful to community mobilizers, health professionals and policymakers.

**Objective:**

To assess the prevalence and determinants of hesitancy to COVID-19 vaccination among patients attending public hospitals in South Gondar zone, Ethiopia.

**Methods:**

A multicenter facility-based cross-sectional study was conducted from 1 November to 30 December 2021 to assess the prevalence and determinants of hesitancy to COVID-19 vaccination. Chi-square test and multivariable logistic regression methods were employed using SPSS 23. Significance level was examined using an odds ratio at 95% CI. Multi-collinearity and model fitness were also checked.

**Results:**

A total of 415 participants were included in the study, with a questionnaire response rate of 100%. The prevalence of hesitancy to COVID-19 vaccination was 46.02%. Age of >49 years, rural residency, fear of the adverse effects of the vaccines, myths about vaccine ineffectiveness and poor practices in COVID-19 prevention were the most common determinants of hesitancy.

**Conclusions:**

Despite increased global morbidity and mortality due to COVID-19, the prevalence of vaccine hesitancy is still high. Therefore, it is important to create awareness in highly hesitant groups.

## Highlights

• COVID-19 is an ongoing global pandemic
• People are still hesitant about vaccination
• A multicenter facility-based cross-sectional study was conducted
• The prevalence of COVID-19 vaccine hesitancy was 46.02%

## Introduction

The world is currently being strongly affected by the social, economic, psychological and multiple health impacts of this “once in a generation” pandemic [1]. The number of COVID-19 cases has exceeded 170 million, with over 3.8 million deaths reported, and almost all government and private sectors have been affected by the pandemic [2,3]. The risk of morbidity and death is higher in people with chronic diseases such as diabetes mellitus, immune-compromised patients, people with renal conditions, asthmatics and older people. Pregnant women have also experienced adverse perinatal outcomes due to the pandemic [4,5].

As the pandemic is expected to continue, improving immunization coverage in the general population is the mainstay for decreasing the incidence of COVID-19 [6]. Herd immunity in the general population has to be at least 67% for the incidence rate of COVID-19 to decline [7,8].

Vaccine hesitancy is the delay in acceptance, reluctance or refusal of vaccination despite the availability of vaccination services [9,10]. The World Health Organization identified it as one of the top 10 threats to global health in 2019 [10]. Vaccine hesitancy involves complex decision-making processes influenced by a wide range of contextual, individual, group and vaccine-specific factors, including communication and media, historical confluences, religion/culture/gender/socioeconomic, political, geographic barriers, experience with vaccination, risk perception, design of the vaccination program and non-scientific myths [9,11].

Worldwide, hesitancy to be immunized is the most common barrier to reducing the incidence of COVID-19 [12,13]. Prevalence of vaccine hesitancy has been measured at 37% in Kenya [13], 37% in Bangladesh [12], 33% in Egypt [14], 59% in Portugal [15], 6% in Saudi Arabia [16] and 48% in Kuwait [17]. These studies report that advanced age, female sex, unemployment, illiteracy, low income, rural residence, absence of trust in the health care system, thinking of yourself as in a non-affected group, fear of adverse effects, lack of adequate clinical trials, and lack of trust on vaccine efficacy are significant determinants of hesitancy to COVID-19 vaccination.

Ethiopia is one of the countries where it has been difficult to combat the pandemic [18,19]. The prevalence of vaccine hesitancy has been recorded at 54.5% in the Wolaita zone [20], 45.9% in Amhara regional state referral hospitals [18] and 57.7% in Debre Tabor comprehensive specialized hospital [19]. Despite the shocking economic crisis and the number of deaths due to COVID-19 in Ethiopia, hesitancy to be immunized is still high, even among health professionals who are expected to create awareness in the rest of the population [19,21]. Hence, our study aimed to identify why there are still barriers to vaccination despite the government's efforts to lower COVID-19 incidence and the death rates due to the pandemic in South Gondar zone, Ethiopia.

## Methods and materials

### Study design and setting

A multicenter institutional-based cross-sectional study was conducted among patients attending selected public hospitals in South Gondar zone, Ethiopia, from 1 November to 30 December 2021. South Gondar zone is 1 of 10 administrative zones in the Amhara region. The town is approximately 669 km northwest of Addis Ababa, the capital city of Ethiopia, and 97 km southwest of BahirDar, the capital city of the Amhara region, and it has an elevation of 2706 m above sea level. Addis Zemen Hospital, Nefas Mewcha Hospital, Mekaneyesus Hospital and Ebnat Hospital were selected randomly among the 8 public hospitals of South Gondar zone.

### Study population

Patients aged >18 years who came to the selected hospitals of South Gondar zone, Ethiopia, during the study period were the study population. Patients who were critically ill and/or who had medically known contraindications for the vaccine were excluded from the study.

### Dependent variable

Hesitancy to COVID-19 vaccination.

## Independent variables

Sociodemographic and health-related characteristics.

### Sample size determination and sampling procedure

The sample size was calculated as 415. The sample size was calculated using the assumption of single population proportion formula considering the prevalence of vaccine hesitancy in Debre Tabor (57.7%) [19]; 95% CI, margin of error 5%, 10% non-response rate. Systematic random sampling was employed to recruit study participants after assessing the case flow at each study hospital. The sampling interval was determined by dividing the number of cases over 2 months in each hospital, and the final kth value was 4.2 (the average of all the sites). Hence the first case to come was taken as participant 1, and every fourth case was selected.

### Data collection procedures

Data was collected by 4 midwives using structured questionnaires after training was given for a day in each hospital. The questionnaire was prepared in English and then translated into the local language, Amharic, for data collection. Language experts translated it back to English again for consistency and accuracy. The questionnaire was first piloted in 2 primary hospitals outside the study area.

### Data entry and analysis

After manually checking for completeness and consistency, the data were entered using Epi-data version 4.6 software and analyzed using SPSS version 23 software. Then to know the crude association between vaccine hesitancy and its determinant factors, crude odds ratio was calculated with 95% CI. Variables with an odds ratio of ≤0.2 were considered for multivariate analysis. Variables with adjusted odds ratio (AOR) of ≤0.05 were considered to determine the significance of association. Hosmer–Lemeshow goodness-of-fit test was used to check the model fitness; poor fit was considered at values <0.05; it was considered to have multi-collinearity when the variance inflation factor (VIF) was >10.

### Operational definitions

“Hesitancy to COVID-19 vaccination” was defined as when an individual has a delay in acceptance, reluctance, or refusal of vaccination despite the availability of vaccination services [8]. “Acceptance of COVID-19 vaccination” was defined as when an individual has no form of delay in acceptance, reluctance or refusal of vaccination in the availability of vaccination services [5].

The participant's level of knowledge about COVID-19 was reported as “good knowledge” when the study participant correctly responded to ≥80% of knowledge assessment tools and “poor” if <80% [5,8]. The attitude of the participant towards COVID-19 was categorized as “positive” if they responded favorably to ≥80% of the attitude-related items and “negative” if <80% [5,8].

The participant's level of practice of COVID-19 preventive measures was reported as “good practice” if they correctly responded to ≥80% of practice assessment tools and “poor” if <80% [5,8].

## Results

### Sociodemographic characteristics

A total of 415 participants were included in the study, with a questionnaire response rate of 100%. The majority of participants were aged 30−49 years (48.43%), female (52.05%), orthodox Christian (68.91%), rural residents (75.18%), married (73.25%), had no formal education (51.81%), housewife/farmer (48.19%) and had no childhood immunization (52.53%) (Table 1).

**Table 1:** Socio-demographic and health related characteristics of the respondents at public hospitals in south gondar zone, north west Ethiopia, 2021 (N=415).

Variables	Frequency	Percent
**Age in years**		
29	112	26.99
30-49	201	48.43
>49	102	24.58
**Sex**		
Male	199	47.95
Female	216	52.05
**Religion**		
Orthodox christian	286	68.91
Muslim	112	26.99
protestant	17	4.10
**Residency**		
Rural	312	75.18
Urban	103	28.82
**Marital status**		
Single	93	22.41
Married	304	73.25
Divorced	18	4.34
**Educational status**		
No formal education	215	51.81
Primary	106	25.54
Secondary	72	17.35
Higher	22	5.30
**Occupation**		
Student	74	17.83
Housewife/farmer	200	48.19
Merchant	95	22.89
Government employee	45	10.84
**Monthly income in ETB**		
< 1000	94	22.65
1001-3000	178	42.89
3001-5000	123	29.64
> 5000	20	4.81
**Use of public medias**		
Yes	198	47.71
No	217	52.29
**Has school age child**		
Yes	328	79.04
No	87	20.96
**Childhood immunization**		
Yes	197	47.47
No	218	52.53
**Household number**		
Two	89	21.45
Three to four	198	47.71
>= five	128	30.84
**Chronic illness**		
Yes	25	6.02
No	390	93.08

ETB- Ethiopian Birr.

### Knowledge, attitudes, preventive measures and vaccine hesitancy

The majority (79.28%) of the participants were knowledgeable about COVID-19 vaccines. In addition, most (58.31%) had a positive attitude towards COVID-19 vaccination; surprisingly, the majority (68.92%) had a poor practice of COVID-19 preventive measures. Almost half of the participants (46.02%) were hesitant to be vaccinated against COVID-19 (Table 2). The most common reasons for hesitancy were fear of adverse effects (69.63%), believing that COVID-19 is not fatal (67.54%), believing that the vaccines can be deadly (53.92%) and inadequate data about the vaccines (65.97%) (Table 3). After adjustment for possible confounding variables, age >49 years (AOR 0.56 [0.01−0.73]), rural residency (AOR 2.02 [1.20−3.71]), fear of the adverse effects of the vaccines (AOR 2.23 [1.65−3.21]), myths about vaccine ineffectiveness (AOR 1.52 [1.09−3.07]) and poor practice of COVID-19 preventive measures (AOR 4.76 [2.55−6.97]) were the common determinants for hesitancy to COVID-19 vaccinations (Table 4).

**Table 2:** knowledge of respondents about COVID-19 and its preventive methods in south gondar zone,Ethiopia, 2021 (N=415).

Measurements	Frequency	Percent
**Hesitate to take COVID-19 vaccine**		
Yes	191	46.02
No	224	53.98
**Knowledge level about COVID-19 pandemic**		
Knowledgeable	329	79.28
Not knowledgeable	86	20.72
**Attitude on COVID-19 pandemic**		
Positive attitude	242	58.31
Negative attitude	173	41.69
**Practices on COVID-19 preventive measures**		
Good practice	129	30.08
poor practice	286	68.92

COVID- Corona virus Disease.

**Table 3:** Reasons for Non-Acceptance of COVID-19 Vaccines Among Respondents in south gondar zone, northwest Ethiopia, 2021 (N=191).

Reasons	Frequency	Percentage
Inadequate data about the vaccines	126	65.97
Fear of adverse effects	133	69.63
Think of vaccine being ineffective	94	49.21
Prefer other ways of protection	38	19.89
COVID-19 is not fatal	129	67.54
High chance recovery from COVID-19	47	24.61
Think of the vaccine as a trial	68	35.60
Not comfortable with my age	23	12.04
Vaccine will kill me	103	74.87
Am young and COVID-19 will not kill me	112	58.64

COVID- Corona virus Disease.

**Table 4:** Factors associated with hesitancy to take COVID-19 vaccine at south gondar zone, northwest Ethiopia 2021 (N=415).

Variables	Hesitancy to COVID-19 vaccine		COR*(95%CI)	AOR*(95%CI)
	Yes	No		
**Age in years**				
18-29	59	53	1.32(0.09-3.43)	1.15(0.02-2.94)
30-49	92	109	*ref*	*ref*
>49	40	62	0.76(0.04-0,72)	0.56(0.01-0.73)*
**Sex**				
Male	74	125	*ref*	*ref*
Female	117	216	0.91(0.42.1.28)	0.35(0.29-1.62)
**Educational status**				
No formal education	109	106	1.48(1.21-3.68)	1.21(0.54-2.74)
Had formal education	82	118	*Ref*	*ref*
**Residence**				
Rural	157	155	2.06(1.34-4.70)	2.02(1.20-3.71)⁎⁎
Urban	34	69	*ref*	*ref*
**Household size**				
Two	36	53	*ref*	*ref*
Three- four	94	104	1.33(0.15-2.43)	1.12(0.43-2.22)
> four	61	67	1.34(0.32-3,14)	1.39(0.67-2.54)
**Has school age child**				
Yes	141	187	*ref*	*ref*
No	50	37	1.79(0.14-2.81)	1.32(0.04-1.98)
**Fear of adverse effects**				
Yes	133	108	2.46(1.06-4.21)	2.23(1.65-3.21)⁎⁎
No	58	116	*ref*	*ref*
**Thinking about vaccines effectiveness**				
Effective	97	140	*ref*	*ref*
Not effective	94	84	*1.62(1.17-3.08)*	1.52(1.09-3.07)*
**Knowledge about COVID-19 pandemic**				
Knowledgeable	147	182	*ref*	*ref*
Not knowledgeable	44	42	1.30(0.03-3.12)	1.04(0.01-2.58)
**Attitude about COVID-19 pandemic**				
Positive attitude	102	140	*ref*	*ref*
Negative attitude	89	84	1.45(1.02-3.25)	1.29(0.06-2.07)
**Practices on COVID-19 preventive measures**				
Good practice	27	102	*ref*	*ref*
Poor practise	164	122	5.08(2.03-6,71)	4.76(2.55-6.97)⁎⁎⁎

COVID- Corona virus disease.

⁎ p<=0.05.

⁎⁎ p<=0.01.

⁎⁎⁎ p<=0.001.

## Discussion

The primary findings of our study are the prevalence of hesitancy to COVID-19 immunization and the common determinant factors for hesitancy. The prevalence of hesitancy to COVID-19 vaccination in our study was 46.02% CI (45.03−54.98). This prevalence is comparable with studies in other regions of Ethiopia, Amhara regional state (45.9%) [18] and Wolaita (54.5%) [20], and in Kuwait (47.9%) [17]. Our study found a higher prevalence of vaccine hesitancy compared with studies in Saudi Arabia (6.1%) [16], Egypt (33%) [14], Bangladesh (36.58%) [12] and Kenya (36.5%) [13]. The increased prevalence in our study could be because the majority of our study participants were from rural areas with lower educational levels, an increased number of patients recovered from the pandemic, a lack of trust in the government as well as the health care system, and myths about the vaccines. In addition, most of the studies with lower hesitancy rates were among health professionals rather than the general population, as in our study. A lower hesitancy rate was found in our study compared with studies in Debre Tabor comprehensive hospital, Ethiopia (57.7%) [19] and in Portugal (59%) [22]. One potential explanation for the lower hesitancy rate found in our study is that it was carried out post multiple community mobilizations and reports of many global deaths due to the pandemic and therefore trust in vaccines had increased.

In our study, being aged >49 years decreases the odds of hesitancy to COVID-19 vaccination by 44% (AOR 0.56 [0.01−0.73]). This finding is the reverse of findings elsewhere in Ethiopia, i.e., Wolaita [20], Debre Tabor [19] and Adiss Abeba [23], and in Kenya [13]. A potential explanation is that our study participants believed that the COVID-19 pandemic would affect older age groups more than the side effects of the vaccine; therefore, the fear that older people would be at higher risk of morbidity and mortality from the COVID-19 pandemic meant they were more likely to volunteer to be vaccinated.

Our study found that rural residency increases the odds of vaccine hesitancy 2.02 times (AOR 2.02 [1.20-3.71]). This finding is supported by studies in Kenya [13]. Possible reasons are that rural residents are less educated, have less access to public media, are more prone to non-scientific community myths and delay seeking care. Our study found that fear of the adverse effects of the vaccines increases the odds of hesitancy to COVID-19 vaccination 2.23 times. Studies in southwest and eastern Ethiopia [5,8], Kenya [13] and Kuwait [17] support this finding. One potential explanation for this association could be that people who advocate for the vaccine's adverse effects will prefer not to immunize despite other facts of the pandemic.

Our study found that myths about vaccine ineffectiveness increase the odds of hesitance to COVID-19 vaccination (AOR 1.52 [1.09−3.07]). This finding aligns with other studies in Ethiopia [5,8], as well as in Kuwait [17] and Kenya [13]. One potential explanation for this association is that people who think vaccines are ineffective, still on trial or are religiously not allowed may underestimate the cost of not being vaccinated on their health and productivity.

Lastly, poor practice of COVID-19 preventive measures increased the odds of vaccine hesitancy by 4.76 times. This finding was supported by studies in Wolaita [20] and Northeastern Ethiopia [8], and in China [24]. One potential explanation for this association is that individuals who do not practice preventative measures underestimate the burden of the global pandemic.

### Strengths and limitations of the study

Since the data were collected from different health institutions, it increases the potential generalizability of our findings; however, conducting the study outside health facilities at a community level would further increase generalizability.

## Conclusions and recommendations

Despite increased global mortality and mortality due to COVID-19, the prevalence of hesitance to be immunized against this fatal pandemic is still high. Although different stakeholders are doing their best to increase COVID-19 vaccination coverage incrementally, people still think of the vaccine as ineffective and about adverse effects over its advantage. Younger people, those living in rural areas and those with poor COVID-19 prevention practices were highly hesitant to COVID-19 vaccination. It is important to create awareness in these highly hesitant groups. We recommend that future researchers study hesitancy in the general population outside hospitals.

## Declarations

Consent to participate

Written informed consent from study participants was obtained after thoroughly explaining the aim of the study to each participant.

## Consent for publication

Not applicable.

## Code availability

All data included in this manuscript can be accessed from the corresponding author upon request through the email address. The tool and consent form can be obtained in the supplementary material section.

## Authors’ contribution

Besfat Berihun Erega is the primary author and participated in the conceptualization, design, analysis and interpretation of the data and drafted the manuscript. All co-authors contributed to the design, analysis and interpretation of the data and critically revised the manuscript for important intellectual content. All authors read and approved the final manuscript.

## Author information

1. Besfat Berihun Erega, BSc, MSc in Clinical Midwifery, Lecturer in Debre Tabor University, College of Medicine and Health Sciences, Department of Midwifery.
2. Wassie Yazie Ferede, BSc, MSc in Clinical Midwifery, Lecturer in Debre Tabor University, College of Medicine and Health Sciences, Department of Midwifery.
3. Fillorenes Ayalew Sisay, MSc in Clinical Midwifery, Lecturer in Debre Tabor University, College of Medicine and Health Sciences, Department of Midwifery.
4. Abeba Belay Ayalew, MSc in Clinical Midwifery, Lecturer in Debre Tabor University, College of Medicine and Health Sciences, Department of Midwifery.
5. Gebrehiwot Ayalew Tiruneh, MSc in Clinical Midwifery, Lecturer in Debre Tabor University, College of Medicine and Health Sciences, Department of Midwifery.
6. Erean Shigign Malka, MPH in Bio-statistics and Epidemiology, Lecturer in Selale University, College of Medicine and Health Sciences, Department of Public Health.
7. Habtamu Abie Tassew, MSc in Clinical Midwifery, Lecturer in Debre Tabor University, College of Medicine and Health Sciences, Department of Midwifery.
8. Asrat Alemu, MSc in Clinical Midwifery, Lecturer in Dilla University, College of Medicine and Health Sciences, Department of Midwifery.

## Appendix Supplementary materials

Image, application 1
