# Supplementary material for: COVID-19 vaccine hesitancy in Ethiopia in 2021: a multicenter cross-sectional study
Source: IJID Reg. 2022 Dec 8;6:120–4. doi: 10.1016/j.ijregi.2022.11.006 (PMC9729579; doi:10.1016/j.ijregi.2022.11.006)
Supplement: Supplementary file 1 [file mmc1.docx]

**Annex I.Participant Information Sheet**

**Title of the research**_Hesitancy to coronavirus-19 vaccines is still a hurdle in Ethiopia by 2021, *Multi center cross-sectional study*

**Principal Investigator:** Besfat Berihun (MSc)

**Coordinating Office:** Debre Tabor University, college of medicine and health

science

**Purpose:** The purpose of this study is to assess the Determinants of hesitancy to coronavirus-19 vaccine among clients attending public hospitals of south gondar zone, Ethiopia by 2021

**Confidentiality**: All the information that you provide will be kept confidential.

**Benefit**: You will not get a direct or immediate benefit from the current research, but in the long run the findings might be used by the policy makers **Risk:** The current research does not bring any physical harm, social

discrimination and psychological pain to the participants.

**Inducement, incentive and Compensation:** There is no inducement, incentives or compensations. But we will be much grateful for your participation.

**Results Dissemination:** The result of the study will be disseminated through

publication in scientific journals.

**Right to Refuse or withdraw:** You have the full right not to participate or

withdraw from the study any time.You have also the right not to answer some of the questions that you do not need to respond.

**Person to Contact:** If you have any questions that is not clear about the

research you can contact the principal investigator of this research, Besfat

Berihun with cell phone number of **+251914466141.**

**Annex II: Informed consent form English version**

As to the information given ahead, participating in this study has no risk. Your name will not be written on this form and the information you give will never be shared with others. You may not answer any questions that you do not want to answer and you may end this interview at any time you want. Now I would like to tell you that you are selected randomly to be a participant in the study. Your genuine response to the interviews will be very important for the study. At the same time, we would like to appreciate your voluntary participation in the interview after a thorough understanding of the information given to you.

I have read this form or it has been read to me in the language I realized and

understand all information stated above.

Are you willing to participate in this study?

1. Yes (say thank you, continue)

2. No ( Say thank you leave)

Name of principal investigator: Besfat Berihun

Phone number: +251-921282861

Email: [berihunbesfat@gmail.com](mailto:berihunbesfat@gmail.com)

Name of interviewer__________________signature __________Date_________

Name of data collector _________________signature __________Date_________

Checked by supervisor:

Name ______________________

Signature __________Date________

**Annex III: English Version Questionnaires**

1. **participants socioeconomic and demographic characteristics**

| Number | Question | Response | Response |
| --- | --- | --- | --- |
| 101 | Age | ….……full years |  |
| 102 | Sex | 1. Male 2. Female |  |
| 103 | Highest educational status | 1 Unable to read and write  2. Able to read and write  3. Primary (1-8) completed  4. Secondary (9-12) completed  5. Collage and above |  |
| 104 | Marital status | 1.married  2.Divorced  3.Not married  4.Widowed  99.other specify |  |
| 105 | Ethnicity | 1.Amhara  2.Oromo  3.Tigre  4.Other specify………… |  |
| 106 | Religion | 1.Christian orthodox  2.Protestant  3.Muslim  99.other specify |  |
| 107 | Residency | 1. Urban 2. Rural |  |
| 108 | Occupation | 1. House wife  2. Government employee  3. Merchant  4. Student  5. Other specify |  |
| 109 | Monthly income | ….……….ETB |  |
| 110 | Did you access public medias? | 1. Yes 2. No |  |
| 111 | Is school age child in you home? | 1. Yes 2. No |  |
| 112 | Have you receive any childhood immunization | 1. Yes 2. No |  |
| 113 | Number of family members in your house hold | ….…………. |  |
| 114 | Did you have any diagnosed medical illness | 1. Yes 2.No | If yes go to #115 |
| 115 | If yes for #114 please specify | ….………. |  |
|  |  |  |  |

1. **Respondent's Knowledge, Attitude, and Practice of COVID-19 Vaccine and Its Preventive Measure**

| 1. no | Questions | Response | Remark |
| --- | --- | --- | --- |
| **Knowledge assessment questions** | | | |
| 201 | Is COVID-19 a serious disease ? | 1. Yes 2. No 3. I don’t know |  |
| 202 | Do you know that COVID-19 results a complication ? | 1. Yes 2. No 3. I don’t know |  |
| 203 | Can vaccines effectively prevent COVID-19? | 1. Yes 2. No 3. I don’t know |  |
| 204 | Can COVID-19 be acquired after full vaccination ? | 1. Yes 2. No 3. I don’t know |  |
| 205 | Did you know where COVID-19 vaccines are available ? | 1. Yes 2. No | If yes go to #206 |
| 206 | Where can we get COVID-19 vaccines? | ….………. |  |
| 207 | Do COVID-19 vaccines had side effects? | 1. Yes 2. No 3. I don’t know |  |
| 208 | Are older and chronically diseased persons at increased risk of COVID-19 ? | 1. Yes 2. No 3. I don’t know |  |
| 209 | How COVID-19 be transmitted ? | 1. Poor hygiene 2. Inhalation of the virus 3. Touching contaminated surfaces, eye, ear or mouth 4. Touching others hand,shaking and kissing 5. Other specify |  |
| **Attitude assessment questions** | | | |
| 210 | Did you believe in the health care system of Ethiopia | 1. Yes 2. No |  |
| 211 | Did trust COVID-19 vaccines effectiveness | 1. Yes 2. No |  |
| 212 | Did you fear COVID-19 vaccines | 1. Yes 2. No | If yes go to #213 |
| 213 | Why do you fear COVID 19 vaccines | ….…….. |  |
| 214 | Did you plan to be vaccinated for COVID-19 | 1. Yes 2. I want to delay 3. Not at all |  |
| 215 | Did you refuse to get vaccinated | 1. Yes 2. No | If yes go to # 216 |
| 216 | Reasons for vaccine hesitancy | ….…… |  |
| 217 | What alternative preventive techniques did you apply for COVID-19 ? | ….…….. |  |
| **Practice assessment questions** | | | |
| 218 | Did you practice COVID-19 prevention methods? | 1. Yes 2. No | If no escape #219 |
| 219 | If yes, what preventive methods did you apply ? | ….……… |  |
